# Association of Body Image, Body Weight and Social Media Use: A Narrative Review of Observational and Experimental Evidence of the Last Decade

**DOI:** 10.3390/bs16030422

**Published:** 2026-03-14

**Authors:** Maria Mentzelou, Sousana K. Papadopoulou, Exakousti-Petroula Angelakou, Ioanna P. Chatziprodromidou, Constantinos Giaginis

**Affiliations:** 1 Department of Food Science and Nutrition, School of the Environment, University of the Aegean, 81400 Myrina, Greece; maria.mentzelou@hotmail.com (M.M.); pangelakou@fns.aegean.gr (E.-P.A.); 2 Department of Nutritional Sciences and Dietetics, School of Health Sciences, International Hellenic University, 57400 Thessaloniki, Greece; 3 Medical School, University of Patra, 26504 Patra, Greece; ioannachatzi@med.upatras.gr

**Keywords:** body image, social media use, body satisfaction, BMI, weight concerns

## Abstract

Background/Objectives: The multifaceted concept of body image (BI) refers to an individual’s attitudes and impressions of their body. Negative BI is associated with a number of harmful health consequences, including obesity, eating disorders, and symptoms of sadness. The contemporary digital era, marked by the dominance of platforms, has brought about a considerable transformation in the landscape of BI issues. This study’s goal is to compile and assess the connections between social media (SM) use, body weight, and BI in adult populations. Methods: This is a narrative review that comprehensively searches across multiple academic databases, such as PubMed, Medline, Scopus, Web of Science, and Google Scholar. Studies that used SM (online blogs, microblogs, content communities, or social networking sites) for engagement (e.g., sharing, commenting, liking) or image-related activities (e.g., viewing, posting, or engaging with images) with healthy adults (aged 18–70 years) of any body mass index (BMI kg/m^2^) met the inclusion criteria. Included were observational and experimental studies that examined habitual SM use. Only peer-reviewed works published in English between 2015 and 2025 met the search criteria. Results: The currently available findings suggest that obese people are more dissatisfied with their bodies than people of normal weight, and obese women are more dissatisfied with their bodies than their peers of normal weight. Furthermore, experimental studies have demonstrated that immediate BI is adversely affected by acute exposure to idealized social media photographs. Conclusions: Policies should support specialized training that emphasizes a holistic approach to health and puts functionality and health above attractiveness. This training is crucial for dispelling weight-related stigmas and enabling healthcare providers to offer compassionate treatment that supports mental and physical health. Future research must concentrate on internalization and social pressure or reinforcement because these subjects have not gotten as much emphasis in prior studies. Such mechanism research could help better contextualize the role of recently introduced SM items.

## 1. Introduction

The multifaceted concept of body image (BI) refers to an individual’s attitudes and impressions of their body. Beliefs and thoughts about appearance and body shape are covered by the cognitive component; identification and estimation of body size, shape, and weight are covered by the perceptive component; feelings about one’s body and body (dis)satisfaction are covered by the affective component; and actions taken to change, conceal, alter, or tend to the body are covered by the behavioral component (Hosseini & Padhy, 2025).

Previous studies have indicated that individual and cultural characteristics, such as gender, ethnicity, and sexual orientation, are associated with BI in addition to physical correlates like body mass index (BMI) (Dahlenburg et al., 2020). Research shows that body dissatisfaction affects all adult age groups, genders, and racial/ethnic groupings. Depending on the assessment method, women are more likely than men to experience body dissatisfaction, with prevalence percentages ranging from 13 to 32% vs. 9 to 28% for men (Eck et al., 2022). Additionally, gender differences exist in BI. Women want to be thin and lean, and they are more unhappy when their bodies are larger than they would like. On the other hand, men tend to like strong bodies (Medeiros de Morais et al., 2017).

According to one’s self-evaluation, one can either have a negative BI by being unhappy with their appearance or a positive BI by loving, appreciating, respecting, and accepting their body (Ahuja et al., 2025; Makarawung et al., 2023). Numerous detrimental health effects, such as depression symptoms, disordered eating and obesity, are linked to negative BI (Murray et al., 2016; Silva et al., 2019). Dieting, binge eating, fasting, calorie tracking, and self-induced vomiting are among the disordered eating behaviors that are more likely to occur in people with negative BI. These behaviors have many detrimental long-term health effects (Cabral et al., 2024).

One of the most popular techniques for evaluating a skewed self-perception of body dimensions and unhappiness with BI is the use of figure rating scales with visual representations of body shapes (Thurston et al., 2022). As with eating disorders (EDs), those with high levels of BI dissatisfaction also have abnormalities in the body schema, an implicit sensorimotor picture of the body’s location in space and activity (Naraindas & Cooney, 2023). Depression, anxiety, low self-esteem, disordered eating, and other serious mental health issues are all directly linked to negative BI, which is an increasing public health concern (Cabral et al., 2024; Naraindas et al., 2023).

People’s behavior has been demonstrated to be impacted by the increasing use of social media (SM), having detrimental impacts on BI and EDs (Rounsefell et al., 2020). The panorama of BI issues has changed significantly in the current digital era, which is characterized by the dominance of platforms like Facebook, Instagram, and TikTok (Vandenbosch et al., 2022). These platforms are becoming active forums for social comparison, identity creation, and cultural diffusion rather than just places to showcase idealized images. A culture of constant comparison is fostered by the constant exposure to carefully chosen and frequently digitally enhanced depictions of the human form, which causes widespread body dissatisfaction and psychological anguish across large segments of the world’s population (Merino et al., 2024).

BI issues and EDs are made worse by social networking sites’ amplification of the promotion of idealized BI in relation to fashion, beauty, diets, and fitness (Wang et al., 2025). Nonetheless, it has been proposed that the media reminds people of their preexisting body dissatisfaction rather than creating it (Gruszka et al., 2022). On social networking sites, people frequently look for advice about diet and exercise habits that can help them manage their weight. BI concerns and EDs are made worse by social networking sites’ amplification of the promotion of idealized body image in relation to fashion, beauty, diets, and fitness (López-Muñóz et al., 2025).

The increasing prevalence of obesity highlights the necessity of evaluating BI disorders in individuals who are overweight in order to potentially stop the development of obesity (Gruszka et al., 2022). Obesity and overweight have a detrimental effect on life quality, raise the risk of chronic illnesses and disorders (hypertension, diabetes, hypercholesterolemia, asthma, among others), and are expensive for healthcare systems (Bibiloni et al., 2017). Since the 1980s, the World Health Organization’s (WHO) European Region has seen a threefold increase in the incidence of obesity, and the number of people affected is still growing at a startling rate, and in the next years, it is expected to soar. It is estimated that 1 billion people worldwide—1 in 7 males and 1 in 5 women—will be obese by 2030 (Bibiloni et al., 2017; Moyon et al., 2024). However, decreasing weight can reduce the hazards associated with obesity; even a 5–10% reduction in body weight has positive health effects, such as lowering blood pressure, LDL cholesterol, triglycerides, and other cardiovascular risk factors (Wing, 2010).

**Figure 1:**
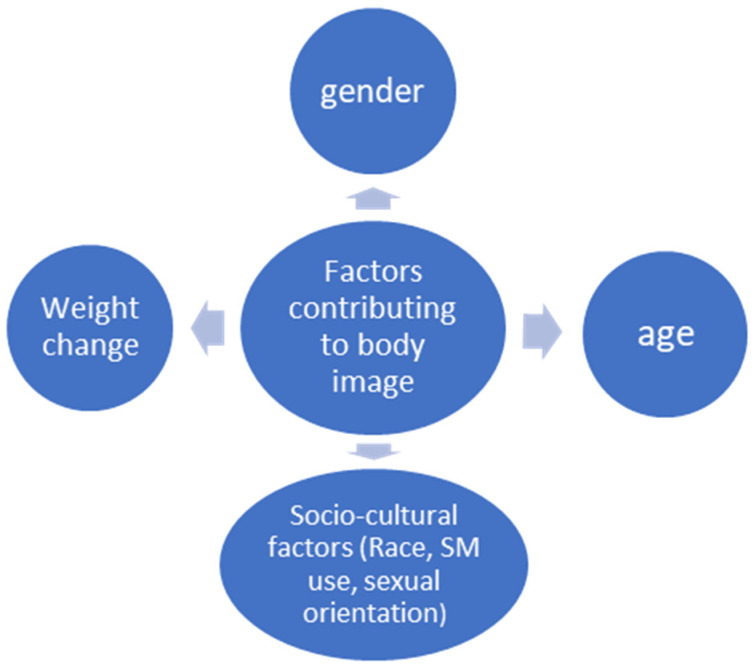
Factors influencing body image.

For example, Sabiston et al. have demonstrated that a person’s assessment of their BI influences their adoption of healthy practices, which in turn impacts the effectiveness of managing obesity (Sabiston et al., 2019). BI is a crucial factor to take into account in the context of obesity when it is affected by an increase in excess weight, especially for women who often have worse BI than males with comparable body types (He et al., 2020). Overall, multiple factors contribute to BI issues (Figure 1).

Existing review literature has addressed BI concerns from related but largely separate perspectives. Reviews focusing on BI and weight or BMI in adults have primarily examined associations with obesity, weight dissatisfaction, and quality of life, often emphasizing clinical or epidemiological outcomes without considering the role of contemporary digital environments (Bibiloni et al., 2017; Moyon et al., 2024; Weinberger et al., 2016). Conversely, reviews examining BI in relation to SM use have predominantly focused on adolescents or young adults and have emphasized media exposure, appearance-based comparison, and eating disorder–related outcomes, frequently without integrating objective or perceived weight status into their conceptual models (de Valle et al., 2021; Merino et al., 2024; Rounsefell et al., 2020; Vandenbosch et al., 2022). Moreover, many existing reviews adopt either a biomedical or a media-effects framework, rather than synthesizing this literature within a shared psychosocial perspective. To our knowledge, no prior narrative review has jointly examined BI in relation to both weight/BMI and SM use across the full adult age range, nor explicitly integrated these domains through common psychological mechanisms such as social comparison, internalization of appearance ideals, stigma, and self-evaluation.

By bridging two previously parallel bodies of literature, the present narrative review seeks to provide a more comprehensive and integrative understanding of how body weight and digital social environments jointly relate to body image in adults. In addition to highlighting the need for comprehensive interventions and policy measures aimed at reducing body dissatisfaction and promoting a healthier and more inclusive conceptualization of body image, this review also explores whether these interacting factors may collectively influence adults’ mental health. Specifically, the review addresses gaps in the literature concerning (1) the relationship between body image and weight/BMI and (2) the relationship between BI and SM use in adult populations, incorporating recent empirical evidence published up to 2024–2025. The originality of this review lies in its focus on adults, a population that has received comparatively less attention in the literature relative to children and adolescents.

## 2. Methods

To conduct this narrative review, a comprehensive search was performed in November 2025 across multiple academic databases, such as PubMed, Medline, Scopus, Web of Science, and Google Scholar. BMI, BI and SM were used in pairs as keywords to collect relevant studies. Moreover, studies that used SM (online blogs, microblogs, content communities, or social networking sites) for engagement (e.g., sharing, commenting, liking) or image-related activities (e.g., viewing, posting, or engaging with images) with healthy adults (aged 18–70 years) of any body mass index (BMI kg/m^2^) met the inclusion criteria. Included were observational and experimental studies that examined habitual SM use. The impact on BI (satisfied or dissatisfaction) or changes in body weight were the outcomes of interest. Only peer-reviewed works published in English between 2015 and 2025 met the search criteria. Studies involving young adults with pre-diagnosed chronic illness, psychological problems, eating disorders (EDs), internet addiction, or engaging in risky health behaviors (such as smoking, heavy drinking, or drug use) were excluded. Studies investigating healthy children and adolescents were also excluded.

To complement the database search, additional strategies were employed, including screening the reference lists of relevant reviews, manually searching key journals, and examining editorials and commentaries. Furthermore, the reference lists of retrieved articles were systematically examined to identify additional relevant studies. All authors participated as reviewers in the screening process. Prior to screening, the reviewers examined the retrieved publications, discussed preliminary findings, and refined the screening and data extraction protocol to enhance consistency and uniformity. Reviewers worked in pairs to assess the titles, abstracts, and full texts of all potentially relevant publications identified through the search strategy. Discrepancies regarding study selection or data extraction were resolved through discussion and consensus. When disagreements persisted, the full team of author-reviewers was consulted to reach a final decision.

**Figure 2:**
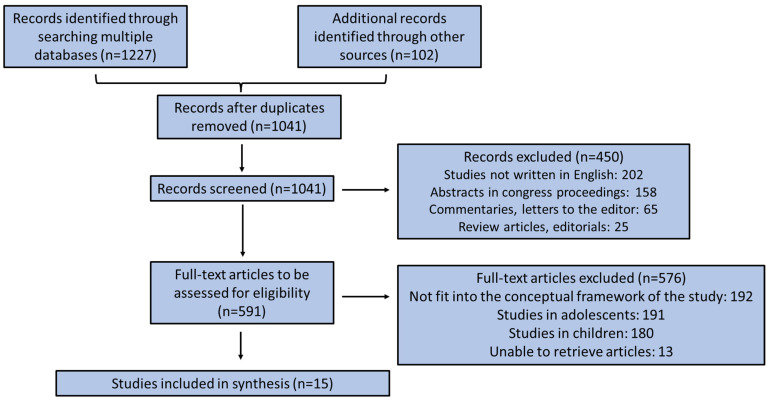
Flow chart diagram of studies’ selection.

Figure 2 illustrates the flow chart diagram of studies’ selection. A total of 1329 studies were generated from the database search, and after removing duplicates, 1041 studies were retained for further evaluation. Through screening titles and abstracts based on inclusion/exclusion criteria, 450 articles were excluded, leaving 591 studies that met the criteria for full-text screening. Among them, 576 full-text articles were excluded for not meeting the inclusion/exclusion criteria, resulting in the final inclusion of 15 studies. As a first part, the studies (n = 8) evaluating the association of BI with body weight were described. As a second part, the studies (n = 7) investigating the association of BM with SM use were presented.

## 3. Results

### 3.1. Body Image and Body Weight

There are several observational and experimental studies in adult populations that evaluated the association between BI and obesity. The currently existing studies are summarized in Table 1.

The goal of Dennis J. S. Makarawung et al. study was to evaluate BI across various weight groups. This study had a cross-sectional design and included the highest number of participants compared to the other relevant studies. It also exclusively included only women. Participants rated the difference between their current and desired body size (BS), as well as their level of happiness with (AE) and investment in (AO) looks. Based on BMI, these scores were compared between weight categories. The questionnaire used self-report to gather information on height and weight, demographics, and level of education. The multidimensional body-self relations questionnaire—appearance scales (MBSRQ-AS) and Stunkard’s figure rating scale (SFRS) were the two questionnaires used in the study’s analysis. The findings showed that there were notable variations between weight groups on every BI scale. Participants in the higher-weight groups generally thought they were less physically attractive, invested less in their appearance, and wanted a smaller body (Makarawung et al., 2023).

Gruszka et al. desired to evaluate the perceptions of body size, weight status, and body dissatisfaction in people who were normal weight, overweight, and obese. For this purpose, a descriptive study was designed in an adequate number of middle-aged participants. Stunkards’ Figure Rating Scale (FRS) was used to measure body dissatisfaction and perceptions of body size. In order to assess participants’ perceptions of their weight status, they were also asked, “Do you think you are: underweight/normal weight/overweight/obese?” After completing the FRS, participants’ height and weight were examined to determine their body mass index (BMI). The distribution of body dissatisfaction by weight in both men and women showed statistically significant differences. Compared to overweight and obese respondents, normal-weight subjects expressed dissatisfaction with their physical size less frequently (Gruszka et al., 2022).

The objectives of another descriptive study were to investigate excess weight (measured and self-reported BMI), BI and satisfaction, self-stigma, positivity, happiness among Spanish individuals who are overweight or obese and the predictors of subjective well-being. In addition to being weighed and having their height assessed, a convenience sample of one hundred overweight people submitted self-reports on the research variables. The participants’ body evaluations revealed, on average, low-to-moderate weight-related stigma, moderate body satisfaction, small excessive weight, and raised positivity and happiness (Godoy-Izquierdo et al., 2020). This study included a smaller number of middle-aged participants compared to the aforementioned studies.

Ibáñez-Zamacona et al., analyzed differences in BI perception and satisfaction by age, sex, and nutritional status in an adult sample from Spain’s Basque Country was the goal of the study. Current Body Image (CBI), Ideal Body Image (IBI), dissatisfaction, and inconsistency scores were assessed using Stunkard’s silhouettes. In an adult population, nutritional status was evaluated using the WHO’s BMI criterion. This is the only case–control study, which included an adequate number of participants. Based on age and sex (early adulthood < 45 years, middle/older adulthood ≥ 45 years), the sample was split into four groups. Dissatisfaction scores revealed a negative correlation between BMI (*p* < 0.001) and sex and age differences (*p* < 0.05). Sex differences in inconsistency scores were only observed in adults aged ≥ 45 (Ibáñez-Zamacona et al., 2020).

In patients undergoing evaluation for bariatric surgery, the aim of the Campedelli’s et al. study was to evaluate the relationships between higher levels of psychopathological aspects, feelings of hopelessness, and psychological and physical health and a high BMI and greater dissatisfaction with BI. This study had a cross-sectional design and included a rather small number of participants compared to the aforementioned studies. The Symptom Checklist-90-Revised, the Body Uneasiness Test, the 12-item Short Form Survey, the Beck Inventory Scale II, and the Beck Hopelessness Scale were completed by 59 individuals undergoing bariatric surgery. There was a considerable correlation between poorer psychophysiological health and dissatisfaction with one’s own BI perception. Conversely, there was no discernible relationship between BMI and the earlier factors. Additionally, a person’s view of their own BI was a major predictor of their psychological wellbeing (Campedelli et al., 2022).

The purpose of Hao’s et al. study was to examine the connections between university students’ nutritional status, eating habits, physical activity, and body dissatisfaction. Additionally, they investigated the viability of raising body dissatisfaction levels in order to improve the nutritional status of university students. Each of the 1900 undergraduate students signed the consent form and offered to take part. Anthropometric assessments and three questionnaires—the Physical Activity Rating Scale-3 (PARS-3), the Chinese version of the Dutch Dietary Behavior Questionnaire (C-DEBQ), and Body Dissatisfaction—were required of the students. Reducing body dissatisfaction has significant potential to prevent obesity, according to the data presented in this study, which highlights the impact of body dissatisfaction among Chinese university students on physical activity deficiency and overeating (Hao et al., 2023). This study had also a cross-sectional design, which was focused on younger adults compared to the other above-mentioned studies.

The Shetty et al. study investigated whether adherence to self-weighing during a 16-week behavioral weight-loss program in 449 obese people was predicted by baseline BMI and BI satisfaction (measured by the Body Image States Scale [BISS]). Throughout the program, participants were given e-scales and urged to weigh themselves every day. The lack of an association between BMI, BISS, and self-weighing was inconsistent with hypotheses and the previous cross-sectional literature, despite results showing negative associations between BMI and BI satisfaction and between self-weighing adherence and weight loss being consistent with previous literature (Shetty & Ross, 2025). Similarly to the above studies, this study also had a cross-sectional design, which exclusively included only middle-aged women.

The study of Argyrides and colleagues investigated the impact of BMI category and gender on a wide range of BI, disordered eating, weight stigma, and psychological well-being factors in a Cypriot population. The Greek version of the Body Appreciation Scale-2 (BAS-2) and the Greek version of the Multidimensional Body-Self Relations Questionnaire—Appearance Scales (MBSRQ-AS) were required by the 642 adults participants. Women who are overweight or obese have consistently reported the lowest results on measures of body image satisfaction and body admiration (Argyrides et al., 2025). This study also had a cross-sectional design, which included an adequate number of middle-aged participants.

The synthesis of evidence regarding BI and weight indicates that while a higher BMI is a robust predictor of body dissatisfaction, the relationship is complex and non-linear. Results across the adult lifespan demonstrate that even ‘normal weight’ individuals report significant body-size non-acceptance, suggesting that BI is heavily influenced by the internalization of appearance ideals rather than objective weight status alone.

A recurrent mechanism identified across studies is weight-related stigma and self-stigmatization, which appears to independently contribute to poorer BI, reduced self-esteem, and psychological distress among individuals with overweight or obesity (Campedelli et al., 2022; Godoy-Izquierdo et al., 2020; Weinberger et al., 2016). Even in samples engaged in weight management or bariatric treatment, BI dissatisfaction is more closely associated with perceived social devaluation than with BMI itself (Campedelli et al., 2022). At the same time, heterogeneity across findings indicates that higher BMI does not uniformly predict negative BI outcomes. Protective factors such as body appreciation, positive self-concept, and supportive social environments have been shown to buffer the negative impact of weight status on BI (He et al., 2020; Moyon et al., 2024), underscoring the need for psychosocial rather than purely weight-centered models.

### 3.2. Body Image and Social Media Use

There are several observational and experimental studies in adult populations that investigated the association between BI and SM use. The currently existing studies are summarized in Table 2.

Czubaj et al., aimed to determine how young individuals’ perceptions of their bodies are impacted by their exposure to fitspiration information on SM. Additionally, to determine whether there are gender differences in the propensity to compare oneself to athletes online and to ascertain how BMI influences the view of one’s physique in relation to SM exposure. The Body Esteem Scale (BES), A. Sobczak’s silhouette scale, which represents various levels of nutrition, and seventeen novel questions about how social media affects BI perception were used in the study. There were 211 responders in the study sample. The necessity for focused treatments to lessen detrimental effects on young adults is highlighted by gender-specific variations in social media-related BI perception (Czubaj et al., 2025). This study had a cross-sectional design, which included relatively young participants. Moreover, the vast majority of participants was women.

The goal of another cross-sectional study was to examine the connections between self-esteem, the propensity to make physical comparisons, and contentment with one’s BI and the amount of time spent on Instagram each day as well as the kinds of content viewed. They recruited 585 participants between the ages of 18 and 40 for the cross-sectional study. The self-esteem scale by Rosenberg, the Physical Appearance Comparison Scale-Revised (PACS-R), the Body Shape Questionnaire (BSQ), and a questionnaire developed by the research team especially for this study that gathered sociodemographic information and Instagram use variables comprised the assessment tools. The study’s findings suggest that using Instagram is linked to lower levels of self-esteem and BI satisfaction, which is mediated by the inclination to compare one’s physical attractiveness to the amount of time spent on the platform each day (Alfonso-Fuertes et al., 2023). However, it should be noted that the vast majority of participants was women, and only a small percentage of 13.7% were men.

The effects of watching “What I Eat In A Day” TikTok videos on American adults were studied by Drivas and colleagues. Throughout the day, participants were randomly assigned to watch films of people consuming more or fewer calories (operationalized in a pilot study as “diets [that]… would lead to weight loss” or “weight gain,” p. 5). Instead, than looking at the direct impact of video type on eating or BI variables, these researchers evaluated a model that looked at the indirect effects of video type on dieting and BI through emotion and social comparison factors. They discovered that the type of video (i.e., watching days with more calories) had an indirect and positive effect on diet goals and body appreciation, and an indirect and negative effect on body dissatisfaction (i.e., reduced body dissatisfaction) (Drivas et al., 2024). In contrast to other studies, this survey had an experimental design and included young women.

Malloy et al.’s study intended to investigate relationships between eating habits, diet quality, and BI disturbance in light of the growing exposure to social media, which frequently promotes unattainable beauty standards. 50 participants were given the Body Image Disturbance Questionnaire (BIDQ) to measure body image disturbance, and the Australian Recommended Food Scores (ARFS) were used to assess diet quality. Additionally, eating behaviors such as emotional eating, uncontrolled eating, and cognitive restriction were evaluated using the Three-Factor Eating Questionnaire (TFEQ-R18). To gauge the impact of social influence, a social influence questionnaire (SIQ) was used. SM’s impact on eating habits and diet quality exhibits minimal correlations, indicating that other factors may mediate these consequences, even though it is linked to BI issues. These findings point to the intricacy of the relationships between eating habits, BI, and diet quality, suggesting that therapies should take SM use into account in addition to the psychological factors that underlie these issues (Malloy et al., 2024). This study had a cross-sectional design and included only a small number of young women.

In order to compare patterns in SM use, BI, and disordered eating behaviors between samples, including as a result of the COVID-19 pandemic, and to test the proposed moderating role of particular content consumed in the association between SM use and maladaptive outcomes, a cross-sectional study surveyed two demographically diverse undergraduate student cohorts in 2015 and 2022. This study included both male and young adults. In 2022, participants reported more frequent vomiting and laxative usage, more BI issues, and more time spent on many SM accounts, with a notable increase in the use of picture-based platforms like YouTube, TikTok, and Snapchat. In particular, exposure to content about weight loss was linked to decreased body appreciation, increased anxiety about being judged negatively, and increased frequency of binge eating (Sanzari et al., 2023). The impact of SM on adult body image is primarily driven by appearance-based social comparison, with findings suggesting that the *type* of content consumed is a more significant predictor of BI disturbance than the total duration of exposure. Specifically, exposure to idealized ‘fitspiration’ or weight-loss content is consistently linked to increased self-criticism and BI disturbance, particularly among women. However, the literature reveals an important nuance regarding content authenticity: experimental evidence suggests that exposure to non-idealized content (e.g., higher-calorie ‘What I Eat In A Day’ videos) can serve as a protective factor, potentially improving body appreciation and reducing dissatisfaction by providing more realistic comparison targets.

In the study of Cossu et al., 129 women saw clean eating or “foodie” content (control condition) on Instagram profiles for 5 min. Positive and negative emotions, as well as body satisfaction, were assessed before and after. Body Image States Scale (BISS) was used to measure individuals’ self-evaluation of and affect regarding their physical appearance. Although there was no statistically significant change in body satisfaction as a simple main effect, the observed time-by-condition interaction indicates that clean eating exposure may have a negative impact on BI (Cossu et al., 2025). This study had a randomized, experimental design, which exclusively included only women.

The cross-sectional survey design of Flores Mata investigated how the Instagram use affected the body dissatisfaction and self-esteem. The Rosenberg Self-Esteem Scale (RSES), the Body Shape Questionnaire (BSQ), and several ad hoc items designed to analyze Instagram usage trends were used in young adults 20 to 40 years (N = 95). The results suggested that as Instagram use increases, body dissatisfaction also increases (Flores Mata & Castellano-Tejedor, 2024).

A central explanatory mechanism is appearance-based social comparison, which is amplified in image-centric environments and particularly salient for individuals with pre-existing BI concerns or higher BMI (Alfonso-Fuertes et al., 2023; Sanzari et al., 2023; Vandenbosch et al., 2022). Closely intertwined with social comparison is the internalization of sociocultural appearance ideals, whereby repeated exposure to narrowly defined body standards promotes the adoption of these ideals as personal benchmarks of self-worth (Bi et al., 2024; Wang et al., 2025). However, the reviewed evidence also highlights meaningful variability. Experimental findings suggest that exposure to more authentic or less idealized content—such as higher-calorie or non-curated food-related videos—may attenuate comparison processes and, in some cases, enhance body appreciation (Drivas et al., 2024; Tiggemann, 2022). These results indicate that SM effects on BI are content-dependent and moderated by individual vulnerability factors, including gender, BMI, baseline self-esteem, and psychological resilience (Merino et al., 2024; Vandenbosch et al., 2022).

## 4. Discussion

This narrative review synthesizes evidence on BI in relation to BMI and SM use in adults, showing that BI disturbances are not explained by body weight or digital exposure alone. Instead, findings point to shared psychological mechanisms—including appearance-based social comparison, internalization of sociocultural appearance ideals, weight-related stigma, and self-evaluation—that help explain variability in BI outcomes across both domains (Alfonso-Fuertes et al., 2023; Bi et al., 2024; Godoy-Izquierdo et al., 2020; Merino et al., 2024; Tiggemann, 2022; Vandenbosch et al., 2022; Weinberger et al., 2016). This integrative view moves beyond fragmented interpretations and highlights BI as a dynamic construct shaped by interacting physical, psychological, and sociocultural factors.

Across the literature, higher BMI is generally linked to greater body dissatisfaction, though this relationship is neither uniform nor inevitable. Evidence suggests that subjective body perceptions, stigma experiences, and self-concept are more strongly associated with BI outcomes than objective BMI alone (Campedelli et al., 2022; Godoy-Izquierdo et al., 2020; Makarawung et al., 2023; Weinberger et al., 2016). Similarly, SM use is not inherently harmful; negative BI outcomes are mainly associated with appearance-focused content that intensifies upward social comparison and ideal internalization (Alfonso-Fuertes et al., 2023; Sanzari et al., 2023; Tiggemann, 2022; Vandenbosch et al., 2022). Conversely, exposure to diverse or less idealized content may mitigate these effects and sometimes promote body appreciation (Drivas et al., 2024; Tiggemann, 2022), highlighting the contextual nature of SM influences.

Overall, associations between BMI, SM use, and BI appear to operate through interconnected psychological mechanisms rather than direct relationships. A key process is appearance-based social comparison, amplified in image-focused SM environments and linked to increased body dissatisfaction following exposure to idealized content (Alfonso-Fuertes et al., 2023; Sanzari et al., 2023; Tiggemann, 2022; Vandenbosch et al., 2022). These comparisons often coincide with the internalization of sociocultural appearance ideals, whereby repeated exposure to thinness- or fitness-oriented norms promotes their adoption as standards for self-worth (Bi et al., 2024; Wang et al., 2025). This dynamic may be particularly detrimental for individuals with higher BMI, as discrepancies between idealized images and lived bodily experiences become more salient, heightening dissatisfaction and psychological distress (Campedelli et al., 2022; Godoy-Izquierdo et al., 2020; Makarawung et al., 2023; Weinberger et al., 2016).

At the same time, weight-related stigma emerges as a critical contextual factor linking BMI to negative BI outcomes. Experiences of stigmatization—both offline and through SM—reinforce negative self-evaluations and contribute to lower body satisfaction independently of objective weight status (Campedelli et al., 2022; Godoy-Izquierdo et al., 2020; Weinberger et al., 2016). These processes are further mediated by self-esteem and self-worth regulation, as individuals increasingly base their self-evaluation on appearance-related feedback, such as likes, comments, and visual engagement metrics on SM platforms (Abrante & Carballeira, 2023; Alfonso-Fuertes et al., 2023). Importantly, not all SM exposure appears uniformly harmful; experimental and observational evidence suggests that content perceived as more authentic or less idealized may attenuate social comparison pressures and foster greater body appreciation (Drivas et al., 2024; Tiggemann, 2022). This variability underscores that it is not merely SM use per se, but the interaction between content type, individual vulnerability (e.g., gender, BMI, pre-existing BI concerns), and underlying psychological mechanisms that shapes BI outcomes (Merino et al., 2024; Sanzari et al., 2023; Vandenbosch et al., 2022).

A positive self-concept and life satisfaction are fostered by a favorable BI, which is a fundamental component of psychological well-being and protects against mental health issues (Merino et al., 2024). About ten years ago, it seemed rather plausible to believe that SM may have a positive impact on BI. Everyone would be roughly satisfied, average bodies would be adjusted, and comparisons would mostly be lateral (or at the very least, any upward comparisons would be balanced by downward ones). However, things have not worked out that way. Specifically, a lot of images of supposedly ideal women with nearly perfect looks and bodies are displayed on photo-based platforms like Instagram. In the end, SM helps to make people feel inadequate in their lives and bodies rather than leveling the playing field and fostering emotions of acceptability and normalcy (Tiggemann, 2022). Additionally, acute exposure to idealized SM photos has been shown in experimental research to negatively affect immediate BI (de Valle et al., 2021; Tiggemann, 2022). People may then turn to unhealthy diets in an effort to attain idealized slimmer bodies as a result of a declining BI. SM posts may also lead people to get fixated on eating healthily and mentally classify things as “good” or “bad” (Sahin & Sanlier, 2025). We found notable inconsistencies, such as one study suggesting that viewing certain types of authentic content (e.g., higher-calorie “What I Eat In A Day” videos) could potentially reduce dissatisfaction—a finding that critically challenges the dominant narrative and demands further experimental exploration.

While the present review offers an extensive look at the relationship between BI, body weight, and digital engagement, several methodological constraints should be noted. The search was restricted exclusively to peer-reviewed works published in English, which likely excludes relevant cross-cultural data and contributes to a language bias. Additionally, the inclusion criteria focused on healthy adults aged 18–70, explicitly excluding individuals with pre-diagnosed chronic illnesses, psychological disorders, or eating disorders. This prevents the findings from being generalized to the very populations often most affected by BI disturbance. Furthermore, the review relies heavily on observational and cross-sectional studies, which limits the ability to draw causal conclusions. There is also significant heterogeneity in the instruments used across the included studies—ranging from Stunkard’s figure rating scales to the Multidimensional Body-Self Relations Questionnaire—which makes direct comparisons between results difficult. For instance, the diversity of BI assessment tools—including instruments such as the Multidimensional Body-Self Relations Questionnaire (MBSRQ), Body Shape Questionnaire (BSQ), Body Appreciation Scale (BAS-2), Figure Rating Scales, Appearance Comparison Scale (ACS), and Sociocultural Attitudes Toward Appearance Questionnaire (SATAQ)—may contribute to methodological heterogeneity, potentially limiting comparability across studies. The above emphasized the need to use a unique BI assessment tool in future studies to enhance the comparison validity among the different studies. Moreover, most studies included either exclusively women or a considerable higher percentage of women than men. The number of participants is also considerably different among the existing studies.

Furthermore, the current manuscript does not explicitly address the potential for publication bias, whereby studies reporting non-significant results may have been underrepresented in the analyzed literature. Additionally, much of the available evidence is correlational, which inherently limits the ability to draw definitive causal inferences regarding the relationships between BMI, SM use, and BI. The predominance of cross-sectional designs, coupled with relatively few longitudinal investigations, further constrains causal interpretation. Sample sizes are highly variable and, in some cases, relatively small, while certain populations—particularly young women—are overrepresented, and cultural diversity across studies remains limited. It is also important to acknowledge the potential for bidirectional relationships; for example, individuals with elevated body dissatisfaction may preferentially engage with appearance-focused content, which in turn could exacerbate such concerns. Finally, the potential influence of uncontrolled confounding variables—such as baseline self-esteem, neuroticism, or prior experiences of weight-related stigmatization—warrants more explicit consideration in future research.

The current investigation also provides valuable insights for health policy and practice, suggesting that addressing BI concerns effectively requires complex, multidimensional strategies. These findings have important implications for healthcare professionals, educators, policymakers, and individuals, all of whom play a critical role in fostering environments that support positive body image and overall well-being (Alleva & Tylka, 2021; Merino et al., 2024; Rodgers et al., 2023). More to the point, public policy initiatives could include the regulation of digital content that promotes narrow body ideals, campaigns encouraging body diversity, and efforts to reduce weight-related stigma. In educational settings, interventions such as media literacy programs and strategies aimed at mitigating appearance-based social comparison may be implemented. Within healthcare contexts, professional training to identify and address body dissatisfaction and stigma is essential, alongside digital platform governance measures—such as moderation strategies or algorithmic adjustments—to limit repeated exposure to highly idealized content.

Moreover, there is an urgent need for more experimental/interventional and longitudinal studies to establish causal relationships, moving beyond the correlational design dominating the current literature. This includes investigating the impact of recently launched SM features (e.g., stories, algorithmic curation) and focusing research on understudied mechanisms like positive BI (e.g., body appreciation) as a resilience factor.

Based on the evidence synthesized in this review, there is consistent support for the conclusion that higher BMI is associated with greater body dissatisfaction and that increased exposure to appearance-focused SM content is linked to more BI outcomes, particularly through mechanisms of social comparison and internalization of sociocultural appearance ideals (Makarawung et al., 2023; Vandenbosch et al., 2022; Shetty & Ross, 2025; Alfonso-Fuertes et al., 2023; Flores Mata & Castellano-Tejedor, 2024; Bi et al., 2024). These associations are robust across diverse adult samples and study designs, although predominantly observational in nature. Accordingly, evidence-supported implications for practice include the need for healthcare professionals, educators, and public health practitioners to recognize body dissatisfaction and weight-related stigma as common psychosocial correlates of both obesity and intensive SM use, and to address these factors in prevention and health-promotion strategies (Merino et al., 2024; Weinberger et al., 2016; Godoy-Izquierdo et al., 2020; Sahin & Sanlier, 2025).

Building on these findings, more forward-looking perspectives suggest that policies and interventions may benefit from adopting holistic, stigma-reducing approaches that prioritize body functionality, psychological well-being, and media literacy over appearance-based ideals. While such approaches are theoretically grounded and supported by emerging experimental and conceptual work (Alleva & Tylka, 2021; Bi et al., 2024; Vandenbosch et al., 2022), their effectiveness in adult populations requires further validation through longitudinal and intervention studies. Future research should therefore focus on testing interventions that target internalization, social comparison processes, and self-esteem regulation in digital environments, as well as evaluating the impact of platform-specific features and content moderation practices on body image outcomes (de Valle et al., 2021; Drivas et al., 2024; Tiggemann, 2022). In addition, an integrative conceptual model synthesizing the psychological mechanisms that may underpin the observed associations could provide a useful framework to guide future research and help to clarify the complex interplay between individual vulnerabilities, social media exposure, and body-related outcomes.

## 5. Conclusions

Overall, the findings of this narrative review demonstrate that BI in adults is shaped by the dynamic and interdependent relationship between body weight and SM engagement. Higher BMI is consistently associated with greater body dissatisfaction; however, this association is strengthened and contextualized by exposure to appearance-focused social media content that promotes idealized body norms. SM use, in turn, does not exert a uniform effect on BI but interacts with individual weight status and psychological vulnerability through mechanisms such as appearance-based social comparison, internalization of sociocultural ideals, and weight-related stigma. These reciprocal processes help explain why individuals across the weight spectrum—particularly those with higher BMI—may experience heightened body dissatisfaction in digitally mediated environments. Collectively, the evidence underscores that BI outcomes arise from the convergence of physical characteristics, digital exposure, and psychosocial mechanisms, highlighting the need for integrated prevention and intervention approaches that simultaneously address weight-related stigma and the nature of SM content and engagement.

## Figures and Tables

**Table 1:** Observational and experimental studies in adult populations assessing the relationship between BI and obesity.

Study Type	Country	Study Population	Basic Results	References
Cross-sectional study	Netherlands	27,896 women, 34.2 ± 12.0 years	People with a higher BMI have on average more BI concerns	(Makarawung et al., 2023)
Descriptive study	Poland	744 adults (452 women, 35.9 ± 12.4 years)	25% of all respondents were satisfied with their own body size Normal weight did not accept their own body size The dissatisfaction with body size was more frequent among women than among men	(Gruszka et al., 2022)
Descriptive study	Spain	100 adults (42.03 ± 10.74, 60% women)	Women reported significantly more negative body self-perceptions Participants with obesity reported lower body satisfaction, a significantly more negative BI	(Godoy-Izquierdo et al., 2020)
Case–control study	Spain	227 women and 178 men, aged 18–70 years	BMI is a biological characteristic related to BI satisfaction Dissatisfaction scores showed that both sex and age differences were negatively associated with BMI	(Ibáñez-Zamacona et al., 2020)
Cross-sectional study	Italy	59 adults, age range 19–55 years	The BI dissatisfaction is more common in people with severe obesity	(Campedelli et al., 2022)
Cross-sectional study	China	1714 young adults (age: 18–24 years; men: 933, women: 781)	Obese people were more likely to be dissatisfied with their body shape	(Hao et al., 2023)
Cross-sectional study	United States	449 adults (age = 49.47 ± 11.37 years, 83.52% women)	Higher BMI was associated with lower ratings of body-image satisfaction	(Shetty & Ross, 2025)
Cross-sectional study	Cyprus	642 adults, 18 to 66 years, 364 women (56.7%)	Individuals suffering from overweight and obesity, regardless of gender, reported considerably higher levels of weight/shape-related anxiety and decreased satisfaction with appearance and body acceptance	(Argyrides et al., 2025)

**Table 2:** Observational and experimental studies in adult populations assessing the relationship between BI and SM use.

Study Type	Author (Country)	Study Population	Basic Results	References
Cross-sectional study	Poland	211 young adults, 27 years old 69 men and 142 women	Gender significantly affects how young adults perceive fitspiration content on social media, with women showing greater self-criticism	(Czubaj et al., 2025)
Cross—sectional study	Spain	N = 585 adults, 18–40 years old 86.3% women	Participants who spent more time on Instagram had higher levels of body dissatisfaction	(Alfonso-Fuertes et al., 2023)
Experimental study	United States	n = 316; mean age = 19.8; 62.7% women	Video type (i.e., viewing higher calorie days) indirectly and positively impacted body appreciation and diet intentions, indirectly negatively impacted body dissatisfaction	(Drivas et al., 2024)
Cross-sectional study	New Zeeland	50 women aged 18–24	Social media familiarity was significantly associated with higher BI disturbance	(Malloy et al., 2024)
Cross–sectional study	United States	2015: 359 adults 2022: 267 adults 2015: age: 19.38 (SD 2.87) years 2022: age: 19.05 (SD 1.5) years	The time someone spends on SM, or the breadth of platforms accessed that is associated with BI disturbance	(Sanzari et al., 2023)
Randomized experimental study	Germany	129 women individuals Mean age = 21 years	No statistically significant change in body satisfaction	(Cossu et al., 2025)
Cross–sectional study	Spain	N = 95 20–40 years old	Instagram use, especially when it comes to appearance-focused content, has a significant impact on body dissatisfaction among young people	(Flores Mata & Castellano-Tejedor, 2024)

## Data Availability

Data is available upon request to the corresponding author.
